# Hyperthermia influences fate determination of neural stem cells with lncRNAs alterations in the early differentiation

**DOI:** 10.1371/journal.pone.0171359

**Published:** 2017-02-24

**Authors:** Lei Wang, Yujia Deng, Da Duan, Shuaiqi Sun, Lite Ge, Yi Zhuo, Ting Yuan, Pei Wu, Hao Wang, Ming Lu, Ying Xia

**Affiliations:** 1 Department of Neurosurgery, Affiliated Haikou Hospital, Xiangya School of Central South University, Haikou, Hsinan, China; 2 Department of Neurosurgery, the Second Affiliated Hospital of Hunan Normal University (PLA 163 Hospital), Changsha, Hunan, China; 3 Key laboratory of Protein Chemistry and Developmental Biology of Ministry of Education, College of Life Sciences, Hunan Normal University, Changsha, Hunan, China; University of Texas at Austin Dell Medical School, UNITED STATES

## Abstract

**Background:**

Temperature is an important parameter in the microenvironment of neural stem cells (NSCs); however, little is known about the precise effects of hyperthermia on fate determination in NSCs or the role of long non-coding (lnc)RNAs in this process. This was addressed in the present study using NSCs cultured at two different temperatures.

**Methods:**

NSCs were divided into 37NSC and 40NSC groups that were cultured at 37°C or 40°C, respectively, for 72 h. Neuronal or glial cell differentiation was evaluated by flow cytometry and western blotting. LncRNA expression was detected by quantitative real-time PCR.

**Results:**

The numbers of cells positive for the neuronal marker Tuj-1 and the glial cell marker glial fibrillary acidic protein were higher in the 40NSC than in the 37NSC group. The two groups also showed distinct lncRNA expression profiles.

**Conclusion:**

Hyperthermia promotes neuronal and glial differentiation in NSCs, which involves specific lncRNAs.

## Introduction

Neural stem cells (NSCs) have the capacity to self-renew and differentiate into neural lineages (neurons, astrocytes, and oligodendrocytes) under specific conditions. NSC transplantation is a promising therapeutic strategy for human central nervous system (CNS) disorders. However, this requires a detailed understanding of the mechanisms underlying NSC differentiation. Transplanted NSCs can integrate into host tissue and differentiate into functional cells [1]. NSC fate determination is a complex process that is controlled by intrinsic and extrinsic regulatory mechanisms in a time- and stage-dependent manner [2]. Neurogenesis and gliogenesis are induced via different signals from the surrounding environment [3]; NSC differentiation depends on intracellular signaling as well as regulation of gene expression and metabolism [4].

Temperature is an important parameter in the NSC microenvironment. Hyperthermia maintains metabolism and promotes resistance to infection and healing. It was previously shown that high temperatures increase leukocyte mobility, enhance leukocyte phagocytosis, and increase T cell proliferation [5]. However, the effects of hyperthermia on NSC fate determination is unknown.

Long non-coding (lnc)RNAs play an important role in NSC fate decisions [6] by regulating gene expression at the epigenetic, transcriptional, and post-transcriptional levels [7]. LncRNAs located in brain-specific regions are specifically expressed during the CNS development and neuronal differentiation [8, 9]. We speculated that specific lncRNAs may be associated with NSC differentiation under hyperthermic conditions. To test this possibility, the present study investigated the effect of hyperthermia on NSC fate specification into neurons and glia. We also examined the potential roles of lncRNAs in hyperthermia-mediated regulation of NSC differentiation.

## Materials and methods

### Animals and cell culture

Newborn Sprague-Dawley (SD) rats were obtained from the laboratory animal department of Central South University and the experimental protocol was approved by the ethical committee of Hunan Normal University. The culture of NSCs were undertaken following the methods of Duan and co-workers[10]. The nerborn rats were humanlu killed by cervical dislocation, and then opened the skull and separated cerebral cortex. The cortex tissue vas separated by micrergy and then dissected to single-cell suspension. The cell suspension was maintained in DMEM/F12 medium supplemented with 2% B27, 20 ng/ml EGF and 20 ng/ml bFGF. The cells were incubated at 37°C and 5% CO_2_ and full humidity.

### Antibodies and chemicals

Primary monoclonal antibodies for Neuronal Class III β tubulin (Tuj-1), nestin, glial fibrillary acidic portein (GFAP), and O4 werepurchased from Abcam (England). B27 supplements, poly-L-lysine (PLL), pidermal growth factor (EGF), cytosine arabinoside (AraC), basic fibroblast growth factor (bFGF) were also obtained from Abcam (England); Fetal bovine serum (FBS) was obtained from Hyclone (USA); Dulbecco’s modified Eagle’s medium (DMEM) and Ham’s F-12 nutrient mixture (F12) were purchased from Gibco BRL (USA); The antibody for flowcytomertry were obtained form Abcam(Tuj-1) and BD(GFAP and O4). All the other chemicals used in the study were of AR grade, available locally.

### Experimental groups

The cultured NSCs were divided into two groups: an hyperpyrexia induction group (40NSCs) and a control group (37NSCs). The former was cultured with DMEM/F12 with 1%FBS under 40°Ctemperature, while the latter was cultured with DMEM/F12 with 1%FBS under 37°Ctemperature.

### Immunofluorescence and flow cytometry

Cells were adhereed onto coverslips, washed with PBS three times, and fixed with 90% alcohol. Cells were incubated with the primary antibody overnight at 4°C. The following primary antibodies were used: rabbit anti-nestin (1:1000) for NSCs, anti-Tuj-1 (1:1000) for neurons, anti-GFAP (1:1000) for astrocytes, anti-O4 (1:1000) for oligodendrocytes. Cultures were then incubated with fluorochromecon jugated secondary antibodies for 1 h at room temperature. Images were taken with an fluorescence microscopy (Carl Zeiss Axioskop2 +, Jena, Germany). For surface protein expression, differentiated cells were plated into a test tube (Becton Dickinson,NJ, USA) at a density of 1×10^5^/mL and washed three timeswith wash buffer (0.1% FBS/PBS). The cells were incubated for 40min with saturating concentrations of fluorescent-conjugated monoclonal antibodies Tuj-1, GFAP and O4. After washing, cell fluorescence signals were determined immediately using flow cytometry with a FACS Caliber instrument (Becton Dickinson, CA, USA). The analysis was performed using Cell Quest Software (BectonDickinson, CA,USA)

### Real tine-qPCR

The total RNA was extracted from cells using the acid guanidinium isothiocyanate-phenol-chloroform method with TRIzol reagent (Sigma) and reverse- transcripted for cDNA synthesis with SuperScript III cDNA synthesis kit (Sigma). Each cDNA subpopulation was subjected to polymerase chain reaction amplification using the specific primers. The sense and antisense primers for each marker were as follows: RMST, F:AAGAGCGGGTGACTGATTG,R:CCTGGTGGGTGATGTGAAG; Tuna, F:CGGCAAGTTCAACGGCACA, R:GACGCCAGTAGACTCCACGACAT; Malat1, F:CTTGGCTTGTCAACTGCG,R:CAAGGAATGTTACCGCACC. The PCR products were mixed with a loading buffer (0.25% bromophenol blue, 0.25% xylene cyanol, and 40% sucrose) and separated on 2% agarose gels. The data was analyzed using MxPro QPCR software.

### Statistical analysis

All values are expressed as mean ± SEM. Statistical comparisons were performed in SPSS 16.0. Student’s two-tailed *t* test was used for comparing experimental groups, and a P value <0.05 was considered significant.

## Results

### NSC culture and identification

**Fig 1:**
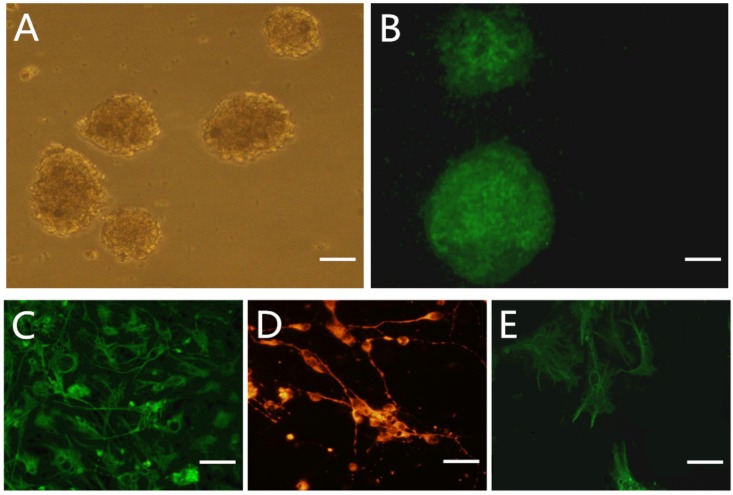
Characterization and differentiation of NSCs. A: phase-contrast image of NSCs globes cultured 5d in NSCs culture medium. B: Immunostaining of NSCs with Nestin antibody. C-F: immunostaining of differentiated cells with astrocyte marker GFAP, neuron marker Tuj-1 and oligodendrocyte marker O4 in 10% FBS-DF_12_ for 5 days. Scale bar: A-B 400 um; C-F 20 um.

NSCs rapidly proliferated in the expansion medium, forming small spheres after 3 days (Fig 1A). On day 5 of culture, neurospheres were positive for the neuronal progenitor marker nestin, as revealed by immunocytochemistry (Fig 1B). NSC spheres cultured in Dulbecco’s Modified Eagle’s Medium containing 10% fetal bovine serum began to differentiate after 2 days. On day 5, the neurospheres had differentiated, as evidenced by increased expression of the neuronal marker Tuj-1 and the glial cell markers glial fibrillary acidic protein (GFAP) and O4 (Fig 1C–1F).

### NSC differentiation

**Fig 2:**
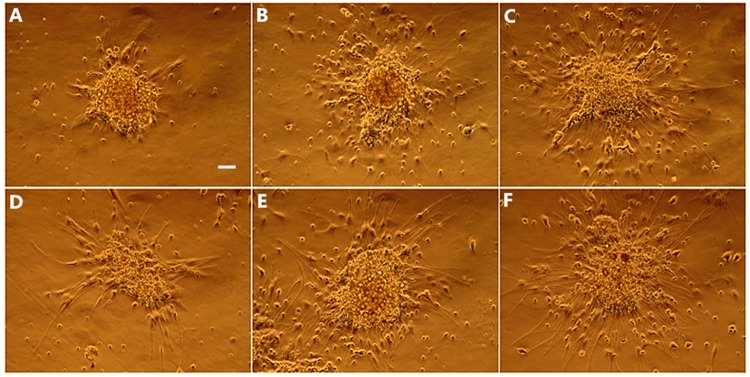
The morphological changes of NSCs at different time point after differentiation. A-C: Morphological changes in 37NSC group during 0h-72h differentiation, most differentiated cells were small, round and triangular or polygonal with 2 to 3 processes. D-F: In 40NSCs group, the differentiated cell exhibited large, flat and elongated shape with longer, wider processes. Scale bar: 100um.

**Fig 3:**
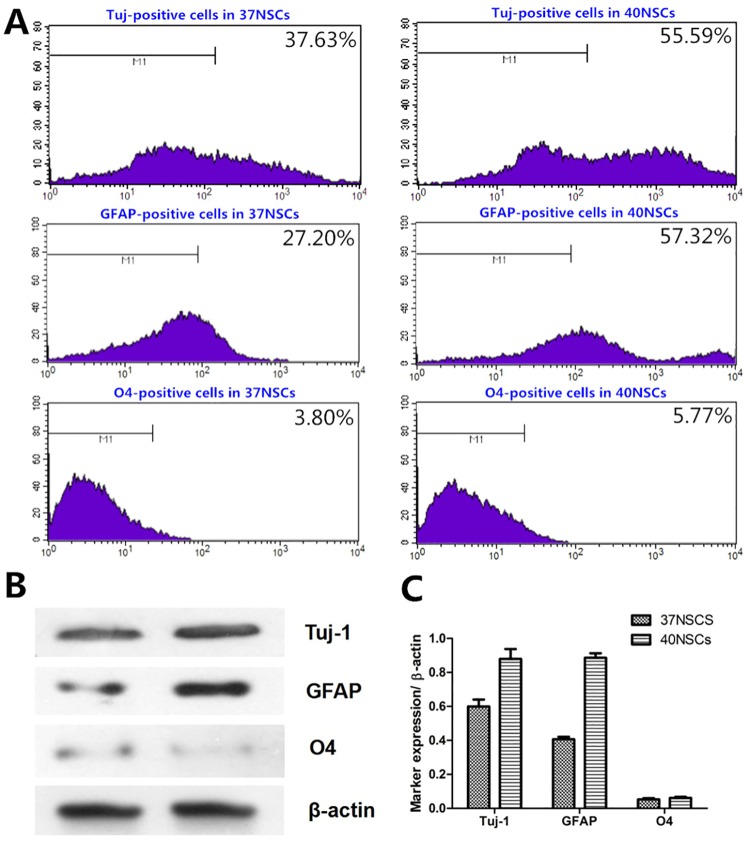
A. Flow cytometric analysis of differentiated cells B. Western blot analysis of Tuj-1, GFAP and O4 of NSCs cultured on 1%FBS-DF_12_ medium of 37NSCs and 40NSCs for 3 days. C. Quantitation of protein bands.*P<0.05.

NSCs cultured in Dulbecco’s Modified Eagle’s Medium supplemented with 1% fetal bovine serum began to differentiate after 24 h. Neurospheres became attached to the dish, and cells grew out of the spheres and assumed an irregular shape (Fig 2A and 2D). Under phase contrast microscopy, a greater proportion of cells were differentiated in the 40NSC than in the 37NSC group after 48 h induction (cultured at 40°C and 37°C, respectively, Fig 2B and 2E). After 72 h, cells in both groups exhibited neuronal or glia-like morphology, with long processes (Fig 2C and 2F). Most differentiated cells in the 37NSC group were small, rounded, and triangular or polygonal with two or three processes (Fig 2C). Cells in the 40NSC group were large, flat, and had an elongated shape with longer and wider processes (Fig 2F). A flow cytometry analysis revealed that the majority of differentiated cells in both groups were positive for Tuj-1 and GFAP (Fig 3A), although there were more cells positive for these markers in the 40NSC as compared to the 37NSC group (55.59% vs. 37.63% and 57.32% vs. 27.20%, respectively) (Fig 3A). O4 was also expressed in differentiated cells, but there was no difference between the two groups in terms of the number of O4-positive cells (Fig 3A). In addition, Tuj-1 and GFAP protein levels were higher in the 40NSCs than in the 37OCM group, as determined by western blotting (Fig 3B and 3C).

### Quantitative Real-Time (qRT-)PCR analysis of lncRNA expression

**Fig 4:**
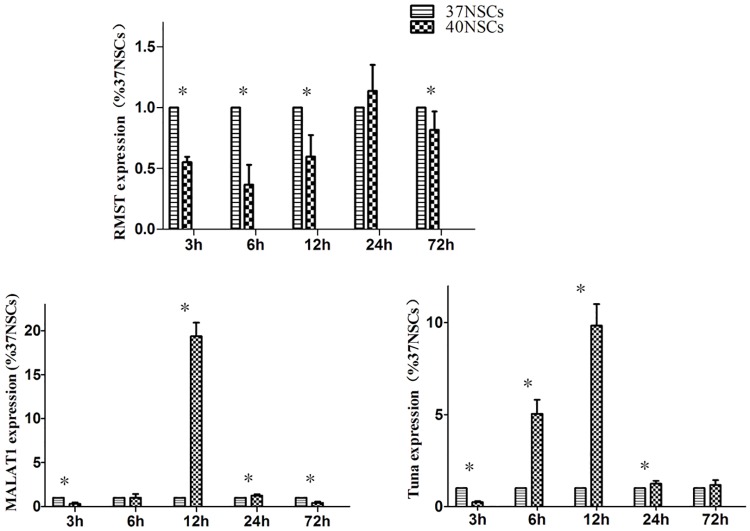
RT-PCR analysis of RMST, MALAT1 and Tuna expression from 0h to 72h after differentiation. *P<0.05.

To investigate the role of lncRNAs in NSC fate specification, we examined the expression of 12 lncRNAs that have been implicated in neuronal/glial differentiation in previous studies [9]. A qRT-PCR analysis detected three lncRNAs—rhabdomyosarcoma 2-associated transcript (RMST), Tuna, and metastasis-associated lung adenocarcinoma transcription (MALAT1)—that were expressed in the 40NSCs and 37OCM groups (Fig 4). Tuna and MALAT1were upregulated in 40NSCs at 12 h, but their levels rapidly declined thereafter. RMST was more abundantly expressed in the 37NSC than in the 40NSC group from 3 to 12 h post-differentiation.

## Discussion

Various factors within the NSC microenvironment affect cell fate, including surrounding cells in the neurogenic niche, the extracellular matrix and basal lamina, and blood vessels [2]. The results of this study demonstrate for the first time that hyperthermia (40°C) is important for promoting NSC differentiation.

Core temperature in humans and other mammals ranges from 36.5°C to 38.5°C according to circadian and metabolic flux [11]. In some cases, the temperature can be as high as 40°C–41°C, for instance during intense exercise, radiation, chemotherapy, and fever. Hyperthermia can confer a survival benefit to organisms [12], and there is evidence in humans of detrimental effects when fever is blocked [13]. Hyperthermia has been shown to promote differentiation in U937 monocytic leukemia [14] and K562 erythroleukemic [15] cell lines. Our findings have important implications for cell-based transplant therapy, since most patients will have varying degrees of fever after NSC transplantation. In addition, stroke and CNS injury are often accompanied by hyperthermia caused by hospital-acquired infection.

We also identified lncRNAs that may modulate hyperthermia-induced differentiation, including RMST, Tuna, and Malat1. RMST is specifically expressed in the brain and is located 150 kb away from the closest annotated protein-coding gene in humans [9]. RNA pull-down experiments have shown that RMST is necessary for neuronal differentiation, while its knockdown inhibited induce glial fate specification with a concomitant decrease in neuronal marker expression [9]. Tuna is associated with pluripotency and neuronal differentiation of embryonic stem cell (ESCs) as well as neurological function in adult vertebrates [16]. Tuna is required for pluripotency and forms a complex with three RNA-binding proteins; knockdown of these proteins or of Tuna suppressed neuronal differentiation in mouse ESCs [16]. Moreover, knockdown of Tuna in zebrafish impaired locomotor function [16]. Malat1 is involved in the recruitment of SR family pre-mRNA splicing factors to the transcription start site; it is highly expressed in neurons and regulates dendritic growth and synapse formation [17] during later stages of neuronal and oligodendrocyte development [18]. Malat1 knockdown decreased synaptic density, whereas its overexpression induced a cell-autonomous increase in synaptic density [17]. Our data indicated that RMST, Tuna, and Malat1 were differentially expressed in NSCs cultured at 37°C vs. 40°C, although the significance of this observation is unclear. It is possible that hyperthermia induces NSC differentiation via regulation of lncRNAs, or that lncRNAs are regulated by another factor and are therefore not directly involved in this process.

Differentiated cells (neurons, astrocytes, and oligodendrocytes) do not survive for long periods when cultured in differentiation medium containing 1% fetal bovine serum due to the lack of essential trophic factors. After 3 days of differentiation, slight vesiculation was observed in neurons and glia appeared enlarged. After 5 days, some differentiated cells began to undergo apoptosis, which was more apparent in the 40NSC group. For this reason we examined cells during a time window of 3 days post-differentiation. Our preliminary experiments showed that Nestin expression declined precipitously after 3 days of differentiation and then decreased gradually from day 3 to 5. This indicated that most of the differentiation process occurs in the first 3 days after induction. A limitation of this study is that the long-term effects of hyperthermia on NSC differentiation could not be investigated.

In summary, we demonstrated that hyperthermia promotes neuronal and glial fate specification in NSCs in the early stages of differentiation, which involves regulation of or by specific lncRNAs. Additional studies are needed to clarify these mechanistic details.

## Abbreviations

CNS: central nervous system
DMEM: Dulbecco’s modified Eagle’s medium
ESCs: embryonic stem cell
GFAP: glial fibrillary acidic portein
LncRNAs: long non-coding RNAs
NSCs: neural stem cells
SD rat: Sprague-Dawley rat
